# Preliminary Observation of the Changes in the Intestinal Flora of Patients With Graves’ Disease Before and After Methimazole Treatment

**DOI:** 10.3389/fcimb.2022.794711

**Published:** 2022-03-25

**Authors:** Mengxue Yang, Xiaodi Zheng, Yueyue Wu, Rui Zhang, Qian Yang, Zhiyan Yu, Jun Liu, Bingbing Zha, Qihai Gong, Bo Yang, Bowen Sun, Miao Zeng

**Affiliations:** ^1^ Department of Endocrinology, Shanghai Fifth People’s Hospital, Fudan University, Shanghai, China; ^2^ Key Laboratory of Basic Pharmacology of the Ministry of Education, Zunyi Medical University, Zunyi, China; ^3^ Joint International Research Laboratory of Ethnomedicine of the Ministry of Education, Zunyi Medical University, Zunyi, China; ^4^ Department of Endocrinology, Zunyi Medical University, Zunyi, China; ^5^ Department of Infectious Diseases I, Shanghai Fifth People’s Hospital, Fudan University, Shanghai, China

**Keywords:** Graves’ disease, intestinal flora, 16 rRNA gene sequencing, Th17 & Treg axis, short chain fatty acids

## Abstract

Immune dysfunction caused by environmental factors plays an important role in the development of Graves’ disease (GD), and environmental factors are closely related to the intestinal flora. Our previous study showed significant changes in the intestinal flora in GD patients compared with healthy volunteers. This study analyzed the relationships between changes in the intestinal flora, thyroid function and relevant thyroid antibodies in GD patients before and after methimazole treatment. The subjects were divided into the UGD group (18 newly diagnosed GD patients), the TGD group (10 GD patients with normal or approximately normal thyroid function after methimazole treatment) and the NC group (11 healthy volunteers). Their fresh stool samples were sent for 16S rRNA gene amplification and Illumina platform sequencing. The correlations of the relative abundance of *Bifidobacterium* with the levels of TRAb, TgAb and TPOAb in the NC group and the UGD group were analyzed. A total of 1,562,445 high-quality sequences were obtained. In the UGD group, the abundances of *Bifidobacterium* and *Collinsella* were higher than that in the NC group; *Bacteroides* abundance in the TGD group was higher than that in the NC group, while *Prevotella* and *Dialister* abundances were lower than that in the NC group; *Prevotella* and *Collinsella* abundances in the UGD group were higher than that in the TGD group. The predominant abundance distribution of Bifidobacteriaceae in the UGD group at the family level was superior to that in the NC group. The abundance of *Bifidobacterium* was positively correlated with the levels of TRAb, TgAb, and TPOAb. The biological diversity of the intestinal flora was reduced in GD patients. After methimazole treatment, the composition of the intestinal flora was significantly altered. The change in *Bifidobacterium* abundance was positively correlated with TRAb, TgAb and TPOAb, suggesting that it might be related to the immune mechanism of GD. The results of this study may deepen our understanding of the pathogenesis of GD and provide a new idea for the treatment of GD.

## 1 Introduction

Graves’ disease (GD) is one of the most common organ-specific autoimmune diseases and accounts for 70%-80% of cases of hyperthyroidism (Chen et al., 2019). Its pathogenesis has attracted much attention from scholars and researchers.

The development of GD is the consequence of the joint action of genetic, immune (including humoral and cellular immunity) and environmental factors (such as infection and intestinal flora imbalance) (Chen et al., 2019; Hou et al., 2021). With genetic susceptibility as a background, the action of initiating factors, such as infection and toxins, induces the variation in the extracellular domain of TSH receptor and immune system dysfunction in the organism, which in turn lead to the functional defect of antigen-specific or nonspecific TS cells and the decline in immune tolerance, recognition and regulation; under such conditions, the organism cannot bring the immune response to its own tissue under control, and TS cells weaken the inhibition of Th cells, while specific lymphocytes promote the B cell production of heterogeneous TRAb with the aid of specific Th cells (Chen et al., 2019). In recent years, systemic research involving animal models of GD and GD patients has provided a new understanding of the development of GD. The intestinal flora may play an important role in the pathogenesis of GD (Su et al., 2020; Hou et al., 2021; Moshkelgosha et al., 2021), and intestinal microorganisms can affect B cells and antibody libraries (Li et al., 2020).

The intestinal flora is composed of more than 1,000 kinds of bacteria, and the approximately 3.8×10^13^ bacterial cells in the body account for 0.3% of body mass; hence, the intestinal flora provides the second largest gene pool in humans and shows heterogeneity and complexity (Eckburg et al., 2005). Studies have shown that the intestinal flora plays an important role in maintaining host nutritional, metabolic, and immune homeostasis. Its composition depends on the interaction of host genetic factors and environmental factors (Arata et al., 2006; Benvenga and Guarneri, 2016). Under normal conditions, the intestinal flora is in a dynamic balance, but it can be affected by factors such as long-term eating habits, drug use, and diseases. When this dynamic balance is disrupted, intestinal flora disorders will occur, and autoimmune reactions are triggered by mechanisms such as molecular simulation, bypass activation, and epitope antigen expansion, leading to autoimmune diseases. Smoking has a proinflammatory effect and can lead to the aggravation of GD and thyroid-associated ophthalmopathy (or Graves’ ophthalmopathy, GO) (Xing et al., 2015; Margret et al., 2019). Stress can cause changes in the levels of intestinal proinflammatory factors and promote the growth and invasion of certain pathogenic bacteria. For example, norepinephrine increases the virulence of bacteria such as Escherichia coli or Campylobacter jejuni and affects the permeability and barrier function of the gastrointestinal mucosa (Lyte et al., 2011), and studies have reported that a vegetarian diet can change the composition of the human intestinal microbiota. A large amount of dietary fiber increases the intestinal flora production of short-chain fatty acids, thereby lowering the pH of the intestine and inhibiting the growth of potentially pathogenic bacteria such as Escherichia coli. Strict vegetarians show a lower risk of hyperthyroidism than omnivores (Zimmer et al., 2012; Tonstad et al., 2015).

Environmental factors leading to changes in the composition and structure of the intestinal flora are related to GD and may play a role in the occurrence and development of GD. In patients with Hashimoto’s thyroiditis, changes in the thickness of the intestinal microvilli at the distal end of the duodenum and an increase in adjacent microvilli spaces have been detected. The permeability of the mucosal barrier increases, and toxins, antigens, bacterial metabolites, and even bacteria undergo translocation, whereby they enter the bloodstream from the intestine and cause continuous inflammation. This triggers autoimmunity and causes autoimmune diseases (Sasso et al., 2004; Mu et al., 2017). Intestinal flora disorders have been shown to play an important role in the occurrence and development of autoimmune diseases such as rheumatoid arthritis, multiple sclerosis, and systemic lupus erythematosus (Chen et al., 2016; Mestre et al., 2019). A previous study conducted by the current team showed that the composition of the intestinal flora was altered in GD patients compared with healthy volunteers, which was particularly manifested by the alterations in the abundances of such genera as Oribacterium, Mogibacterium, Lactobacillus, Aggregatibacter and Mogibacterium (Yang et al., 2019). However, questions regarding whether the findings obtained in that study were a matter of coincidence or were inevitable results and whether thyroid drug (e.g., methimazole) treatment will alter the abnormal composition of the intestinal flora in this population have not yet been settled. Additionally, the association of the alteration of intestinal flora with thyroid function and related antibodies remains unclear.

Based on the aforementioned findings, we enlarged the sample size in this study to further investigate the composition of the intestinal flora in GD patients. The alterations in the intestinal flora were compared before and after methimazole treatment, and the associations of the composition of the flora with thyroid function and related antibodies were also explored. The results of this study might shed new light on the pathogenesis of GD and provide new ideas for the treatment of this condition.

## 2 Subjects and Methods

### 2.1 Subjects

A total of 18 newly diagnosed GD patients who visited the Endocrinology Department of the Affiliated Hospital of Zunyi Medical University from September 2018 to February 2019 were consecutively enrolled in the UGD group (pretreatment), including 7 males and 11 females aged 36.67 ± 11.167 years. Another 10 GD patients receiving 6 months of methimazole treatment (10 mg/tablet, 10-30 mg/day) (Saizhi, Merck Pharmaceutical Jiangsu Co., Ltd.) were included in the TGD group (posttreatment), including 4 males and 6 females aged 30.2 ± 9.69 years, whose serum free triiodothyronine (FT3), free thyroxine (FT4), and thyroid-stimulating hormone (TSH) levels returned to almost normal. In addition, 11 healthy people who went for a health checkup in the Physical Examination Center of the Affiliated Hospital of Zunyi Medical University in the same period were included in the normal control group (NC group), including 5 males and 6 females aged 28.91 ± 10.82 years (Chen et al., 2019). The diagnostic criteria were as follows: newly diagnosed GD patients (UGD group) who were in compliance with the 2008 Chinese Society of Endocrinology “Guidelines for Diagnosis and Treatment of Thyroid Diseases in China” (Chinese Society of Endocrinology and Compiling Group, 2007) and had not received any treatment. The patients with thyroid function approaching or returning to normal levels after methimazole (Saizhi) treatment (TGD group) also had to show parameters in line with the 2008 Chinese Society of Endocrinology “Guidelines for Diagnosis and Treatment of Thyroid Diseases in China” (Chen et al., 2016); had to be taking the antithyroid drug methimazole (Saizhi, Merck Pharmaceutical Jiangsu Co., Ltd.) for treatment and showing relief of hyperthyroidism symptoms; and their serum TSH, FT3, and FT4 levels had to return to almost normal. Inclusion criteria for the NC group were as follows: patients without GD or other organic diseases, with normal routine laboratory test results (blood, urine, and stool routine, liver and kidney function, serum lipids, blood glucose, etc.) and normal imaging examination results (chest radiograph, electrocardiogram, and abdominal color Doppler ultrasound); this group was age- and sex-matched with the GD patients (Hou et al., 2021). The diagnostic criteria for exclusion were as follows: patients who had taken any food or drugs that may affect their intestinal flora, such as antibiotics, Chinese herbal medicines, probiotics, or prebiotic microecological preparations, in the past month; pregnant women or those in the lactation period; alcoholics (drinking more than 5 times a week and consuming an average of 2 taels of white wine or half a catty of rice wine or more than 5 bottles of beer each time); patients with severe mental illness in the past 6 months; patients who had been taking medication for various chronic diseases for more than 1 month in the past 6 months; patients who had been administered medication for the following diseases in the past month: cholecystitis, peptic ulcer, urinary tract infection, infectious diarrhea, acute and chronic sinusitis, tonsillitis, and acute and chronic bronchitis; patients with severe liver diseases such as chronic or persistent hepatitis or cirrhosis; patients with positivity for the hepatitis B virus surface antigen (HBsAg) and abnormal liver function (serum alanine aminotransferase and aspartate aminotransferase levels 2.5 times the normal values); and people suffering from acute or chronic renal insufficiency, severe organic diseases, autoimmune diseases, or infectious diseases such as tuberculosis or AIDS or who had undergone surgery to lose weight.

All subjects voluntarily offered to participate in the study and signed informed consent forms. We also obtained approval from the Ethics Committee of the Affiliated Hospital of Zunyi Medical University (2018) 1-145.

### 2.2 Methods

#### 2.2.1 Medical History Taking

A unified epidemiological questionnaire was adopted, and a dedicated person was engaged in recording the general condition of each patient, including their name, sex, age, hospitalization number or outpatient ID number, diagnosis date, history of present illness, past history, personal history, and family history.

#### 2.2.2 Anthropological Parameter Measurement

Subjects’ height, weight, and blood pressure were measured by a designated person. Body mass index (BMI) = weight/height2 (kg/m2) was calculated. Subjects were asked to take off their shoes and coats and to wear single underwear when measuring their weight. The weight scale was corrected to an accuracy of 0.5 kg, and the height measurement was accurate to 0.1 cm.

#### 2.2.3 Biochemical Indicator Detection

A Siemens Centaur-XP automatic chemiluminescence immunoassay analyzer was employed to detect thyroid function [FT3, FT4, TSH, thyroid stimulating hormone receptor antibody (TRAb), thyroglobulin (Tg), thyroglobulin antibody (TgAb), and thyroid peroxidase antibody (TPOAb)]. A Beckman Coulter AU5821 automatic biochemical analyzer was applied to test blood lipids [triglycerides (TG), total cholesterol (TC), high-density lipoprotein (HDL-C), low-density lipoprotein (LDL-C), apolipoprotein A1 (ApoA1), and apolipoprotein B (ApoB)], fasting blood glucose (FPG), liver function [aspartate aminotransferase (AST), alanine aminotransferase (ALT), and albumin (ALB)], and kidney function [urea (Ur), serum creatinine (SCr), serum uric acid (SUA), and serum cystatin C (SCys-C)]. A Sysmex XE-5000 automatic blood cell analyzer was applied to perform routine blood tests [white blood cell count (WBC), neutrophil absolute value (NEUT#), red blood cell count (RBC), hemoglobin (HGB), and platelet count (PLT)].

#### 2.2.4 Medical Imaging Examination

Carotid color Doppler ultrasound was used to qualitatively diagnose the presence of diffuse goiter, which was performed by the designated sonographer in our hospital, and the diagnosis was made according to the standard criteria.

#### 2.2.5 Stool Specimen Collection

An approximately 10-g fresh stool specimen was collected from each subject at multiple locations by using a special stool sampler and strictly following aseptic concepts. Then, the sample was quickly transferred in an ice box to a -80°C freezer for storage.

#### 2.2.6 Intestinal Flora DNA Extraction

Bacterial genomic DNA was extracted with a DNeasy PowerSoil kit (Mo Bio/QIAGEN), and the extracted DNA was detected. The absorption (A) values of DNA were read at 260 nm and 280 nm with a fluorescence spectrophotometer (NanoDropND-1000; Thermo FisherScience, Waltham, MA, USA). An A260/A280 ratio falling between 1.8 and 2.0 indicated satisfactory purity of RNA, which could be used for the subsequent experiment. The quality of DNA was detected by 1% agarose gel electrophoresis. The DNA concentration was adjusted appropriately. The DNA working solution was stored at 4°C, and the storage solution was stored at -20°C.

#### 2.2.7 Gene Amplicon for 16S rRNA Sequencing

The 16S rRNA sequencing was performed by Shanghai Personal Biotechnology Co., Ltd., Shanghai, China.

Ribosomal RNA (rRNA) contains multiple conserved regions and highly variable regions, and primers were designed according to the conserved regions to amplify a single or multiple variable regions of rRNA genes, which were then sequenced to analyze microbial diversity. Due to the read length limitation of MiSeq sequencing and to ensure the quality of the sequencing results, the best sequencing insert size ranged from 200-520 bp. In this study, the highly variable V3-V4 region of the bacterial 16S rRNA gene with a length of approximately 468 bp was used for sequencing. The bacterial 16S rDNA V3-V4 region-specific primers 338F (5’-barcode+ACTCCTAC GGGAGGCAGCA-3’) and 806R (5’-GGACTACHVGGGT WTCTAAT-3’) were used for PCR amplification. The barcode of the forward primer was a 7-base oligonucleotide sequence used to distinguish different samples in the same library.

#### 2.2.8 Quantification and Mixing of PCR Products

The Quant-iTPicoGreendsDNA Assay Kit was used to quantify the PCR products on a microplate reader (BioTek, FLx800), and then mixing was performed according to the required amount for each sample.

#### 2.2.9 Library Construction

The library was constructed by using the TruSeq Nano DNA LT Library Prep Kit from Illumina Inc. The end repair process was first performed by using End Repair Mix2 in the kit to remove the bases protruding from the 5’ end of the DNA, fill in the missing bases at the 3’ end, and add a phosphate group to the 5’ end. The specific steps were as follows:

(1) Add water to 30 ng of mixed DNA fragments to a volume of 60 μL, followed by the addition of 40 μL of End Repair Mix2;
(2) Mix with a pipette and incubate on a PCR machine at 30°C for 30 min;
(3) The end repair system was purified with BECKMAN AMPure XP beads and finally eluted with 17.5 μL of resuspension buffer.

The second step was to add an A base to the 3’ end. In this process, a single A base was added to the 3’ end of the DNA to prevent the self-linking of DNA fragments, and it was ensured that there was a protruding T base at the 3’ end of the DNA that could be connected to a sequencing adapter. The specific steps were as follows:

(1) Add 12.5 μL of A-Tailing Mix to the selected DNA;
(2) Mix with a pipette and incubate on a PCR machine. The procedures were set as follows: 37°C for 30 min, 70°C for 5 min, 4°C for 5 min, and 4°C for ∞.

The third step was to add an adapter with a specific label. This process was used to achieve the hybridization of the DNA with the flow cell. The specific steps were as follows:

(1) Add 2.5 μL of resuspension buffer, 2.5 μL of ligation mix, and 2.5 μL of the DNA adapter index to the A-primed system;
(2) Mix with a pipette and incubate for 10 min in a PCR machine at 30°C;
(3) Add 5 μL of stop ligation buffer;
(4) The system to which the adapter had been added was purified by using BECKMAN AMPure XP beads.

The fourth step was to amplify the DNA fragments to which adapters had been added by PCR and to then purify the PCR system by using BECKMAN AMPure XP beads.

The fifth step was to perform the final fragment selection and purification of the library through 2% agarose gel electrophoresis.

#### 2.2.10 Library Quality Inspection and Sequencing

(1) Library quality inspection and quantification. The quality inspection for the library was carried out by loading 1 μL of the library into an Agilent 2100 Bioanalyzer and using an Agilent High Sensitivity DNA Kit. A qualified library should have a single peak without an adapter. The quantification of the library was carried out on a PromegaQuantiFluor system by using the Quant-iTPicoGreendsDNA Assay Kit, and the calculated concentration of the qualified library was above 2 nM.
(2) Sequencing. Qualified libraries were subjected to 2×250 bp paired-end sequencing by using a MiSeq Reagent Kit V3 (600 cycles) on a MiSeq machine. First, the library to be detected on the machine (index unrepeatable) was diluted in a gradient to 2 nM, and the samples were then mixed according to the required volume and proportion. The mixed library was denatured into single strands by using 0.1 N NaOH and sequenced on the machine. The amount of the loaded library was controlled to between 15-18 pM based on the actual measurements.

### 2.3 Statistical Analysis

SPSS 22.0 software was applied for the statistical analysis, and GraphPad Prism 5.0 was used for plotting. Normally distributed measurement data were expressed as x ± s, and the independent-samples Student’s t test was used for comparisons between two groups. Nonnormally distributed data are presented as the median (interquartile range), and the Mann–Whitney U test or Kruskal–Wallis H test was used for statistical analysis. For continuous variables, Spearman correlation analysis for nonnormally distributed data was used. Bonferroni correction was used for pairwise comparisons among the three groups, and P<0.017 indicated that the difference was statistically significant. For comparisons between two groups, P<0.05 was considered statistically significant.

## 3 Results

### 3.1 Basic Clinical Features of the Three Groups

The TSH level in the UGD group was lower than that in the NC group, while the FT3, FT4, TRAb, TPOAb, and TgAb levels were all higher than those in the NC group (P<0.017). The TSH level in the TGD group was higher than that in the UGD group, while the FT3 and FT4 levels were lower than those in the UGD group (P<0.017). The differences in the TSH, FT3, or FT4 levels between the TGD and NC groups were not statistically significant (P>0.017). There was no significant difference in sex, age, or BMI among the 3 groups (P>0.017) (**Table 1**).

**Table 1:** The basic clinical features of the three groups.

Clinical indicators	(NC)	GD pretreatment (UGD)	GD posttreatment (TGD)	Reference range
Number of cases	11	18	10	
**Sex**				
Male [n (%)]	5 (46)	7 (39)	4 (40)	
Female [n (%)]	6 (54)	11 (61)	6 (40)	
Age (years)	28.5 (16–47)	39 (25–50)	29.5 (19-48)	
BMI (kg/m^2^)	20.78 (19.6-23.67)	19.92 (18.0-24.05)	21.53 (19.37-23.79)	
**Thyroid hormones**				
FT3 (pmol/L)	5.00 (1.57-5.80)	30.80 (8.40-30.80)*	5.05 (3.80-6.00)#	2.77-6.31
FT4 (pmol/L)	16.90 (12.80-23.70)	71.40 (20.60-154.80)*	14.90 (10.60-19.60)#	10.45-24.38
TSH (μIU/mL)	1.895 (1.009-4.153)	0.005 (0-0.011)*	3.670 (0.096-13.741)#	0.55-4.78
TRAb (IU/L)	0 (0-0.79)	17.82 (5.27-29)*	–	<1.75
TgAb (IU/mL)	0 (0-13.9)	464.9 (10.1-4000)*	–	0-34
TPOAb (IU/mL)	11.9 (7.9-15.3)	107.2 (15.2-600)*	–	0-115

Compared with the NC group: *P < 0.017; compared with the UGD group: #P < 0.017.

Data are presented as the median (IQR), or n (%).

### 3.2 Operational Taxonomic Unit (OTU) Division and Classification

A total of 1,562,445 high-quality sequences were detected by Illumina sequencing. Among these sequences, 477,940 sequences were obtained in the NC group, 722,725 in the UGD group and 361,780 in the TGD group, and each sample contained (40,063 ± 6,355) valid sequences on average. In the field of microbial ecology, 97% sequence similarity was used as the OTU partition threshold, which was roughly equivalent to the taxonomic sequence difference at the species level. The Greengenes database was used for OTU division according to template sequences for the identification of OTU taxonomic status based on the 16S rRNA gene database of bacteria, and statistical analysis of the identification results was subsequently carried out.

### 3.3 Venn Diagram of the Three Groups at the OTU Level

**Figure 1:**
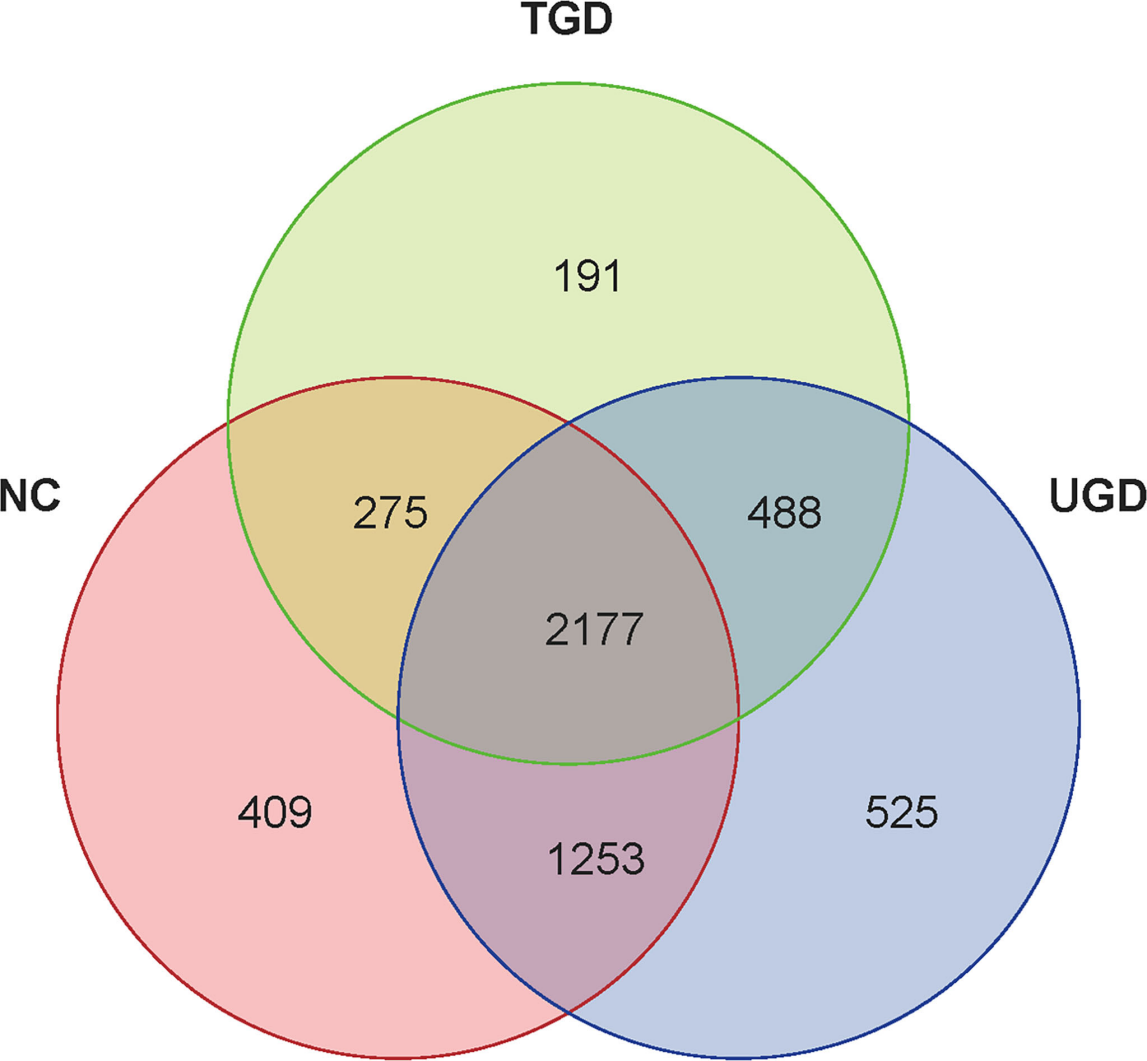
Venn diagram of the three groups at the OTU level. Each circle represents the corresponding group, the overlapping area between the circles indicates the common OTUs between the groups, and the number within each block represents the number of common or unique OTUs contained in the block.

According to the obtained OTU abundance matrix, R software was used to calculate the number of OTUs in the three groups, and the numbers of common and unique OTUs in the three groups were visually displayed in the Venn diagram of OTUs. The NC group exhibited 409 unique OTUs, the UGD group exhibited 525 OTUs, and the TGD group exhibited 191 OTUs. The three groups showed a total of 2,177 common OTUs (**Figure 1**).

### 3.4 Alpha Diversity Analysis of Intestinal Flora Among the Three Groups

**Figure 2:**
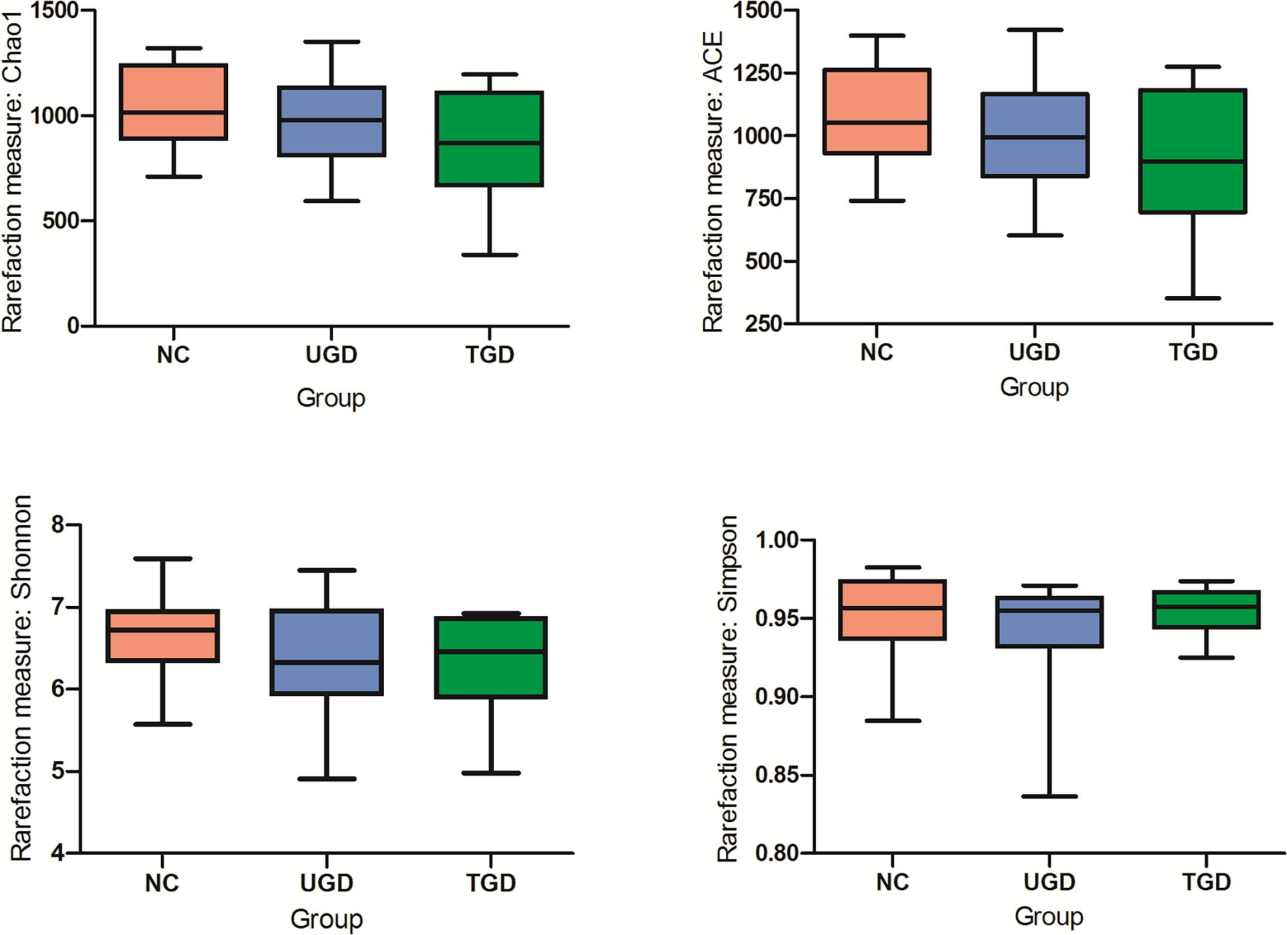
Significance of the alpha diversity estimators among the three groups.

**Figure 3:**
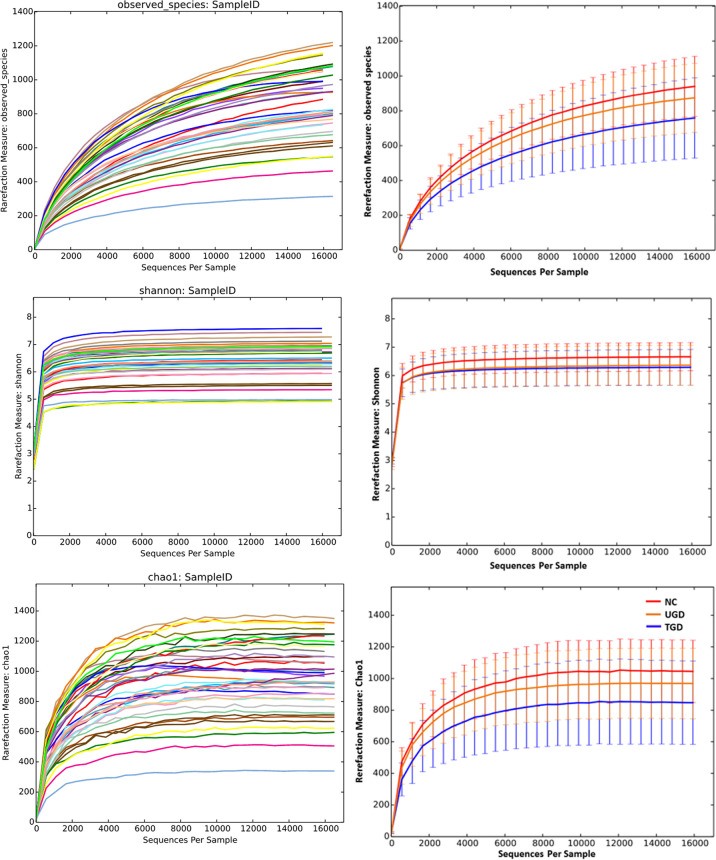
Rarefaction curves of the Chao1 and Shannon indexes among the three groups. The abscissa represents the total number of randomly selected sequences in each sample, and the ordinate represents the number of OTUs observed at the corresponding depth.

Based on the Kruskal–Wallis H test, the Chao1, ACE (focusing on the richness of the community), Shannon and Simpson (considering both the richness and the uniformity of the community) indexes were not significantly different among the three groups (P>0.05) (**Figure 2** and **Table 2**). We further plotted the rarefaction curve. As the sequencing depth increased, the curve gradually plateaued. It was no longer possible to detect a large number of newly undiscovered OTUs with an increasing sequencing depth, indicating that the sequencing results were sufficient to reflect the diversity contained in the current sample (**Figure 3**). Although the Chao1 index, ACE index, Shannon index, and Simpson index showed no significant difference, they were higher in the NC group than in both the UGD and TGD groups, and they were higher in the UGD group than in the TGD group, demonstrating that the biological diversity of the intestinal flora was reduced in the patients with GD.

**Table 2:** The diversity indexes of GD patients before and after treatment.

Diversity index	UGD (before treatment)	TGD (after treatment)	*P*
Chao1	976.725 (594.5-1350.58)	868.975 (339.25-1195.37)	0.38
ACE	993.725 (602.45-1420.06)	895.935 (351.1-1274.66)	0.33
Shannon	6.325 (4.91-7.45)	6.455 (4.98-6.92)	0.25
Simpson	0.955 (0.836-0.971)	0.957 (0.925-0.974)	0.37

### 3.5 Beta Diversity Analysis of Intestinal Flora Among the Three Groups

**Figure 4:**
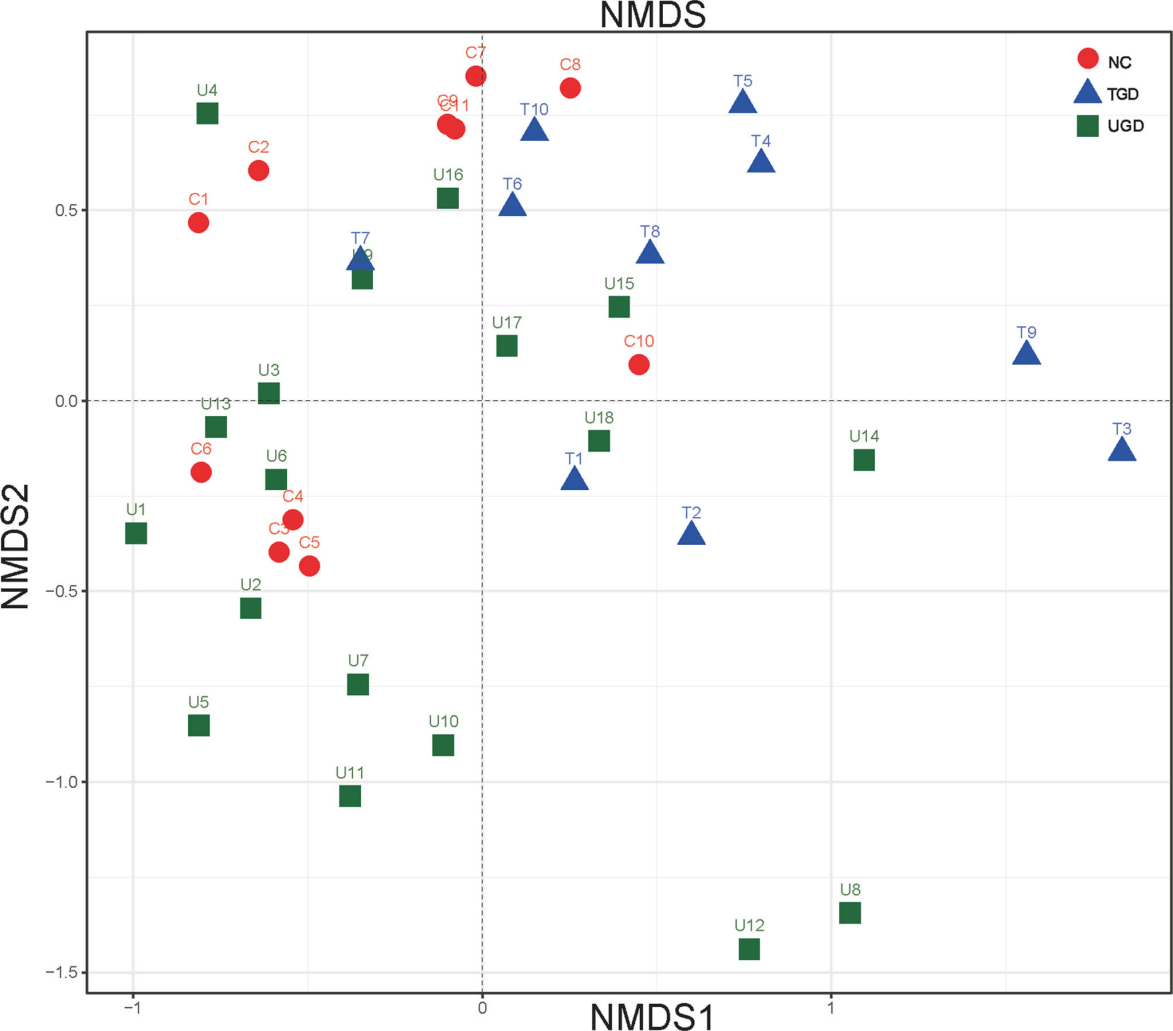
Two-dimensional sorting diagram of samples analyzed by unweighted UniFrac NMDS among the three groups. Each point represents a sample, and points of different colors belong to different groups.

Regarding the beta diversity, by ranking the distances of the samples, the order of the samples in the low-dimensional space was determined to conform to the distance relationships among them to the greatest degree possible; that is, nonmetric multidimensional scaling (NMDS) with unweighted UniFrac distance analysis showed that there were certain overlaps among the three groups, with the NC group samples mainly being distributed in the upper left region, the UGD group samples in the lower left region, and the TGD group in the upper right region. NMDS distinguished the samples in the three groups, and a high similarity of the community structure was found among the samples within one specific group (**Figure 4**).

### 3.6 Difference Analysis of the Intestinal Flora Among the Three Groups at Each Classification Level

**Figure 5:**
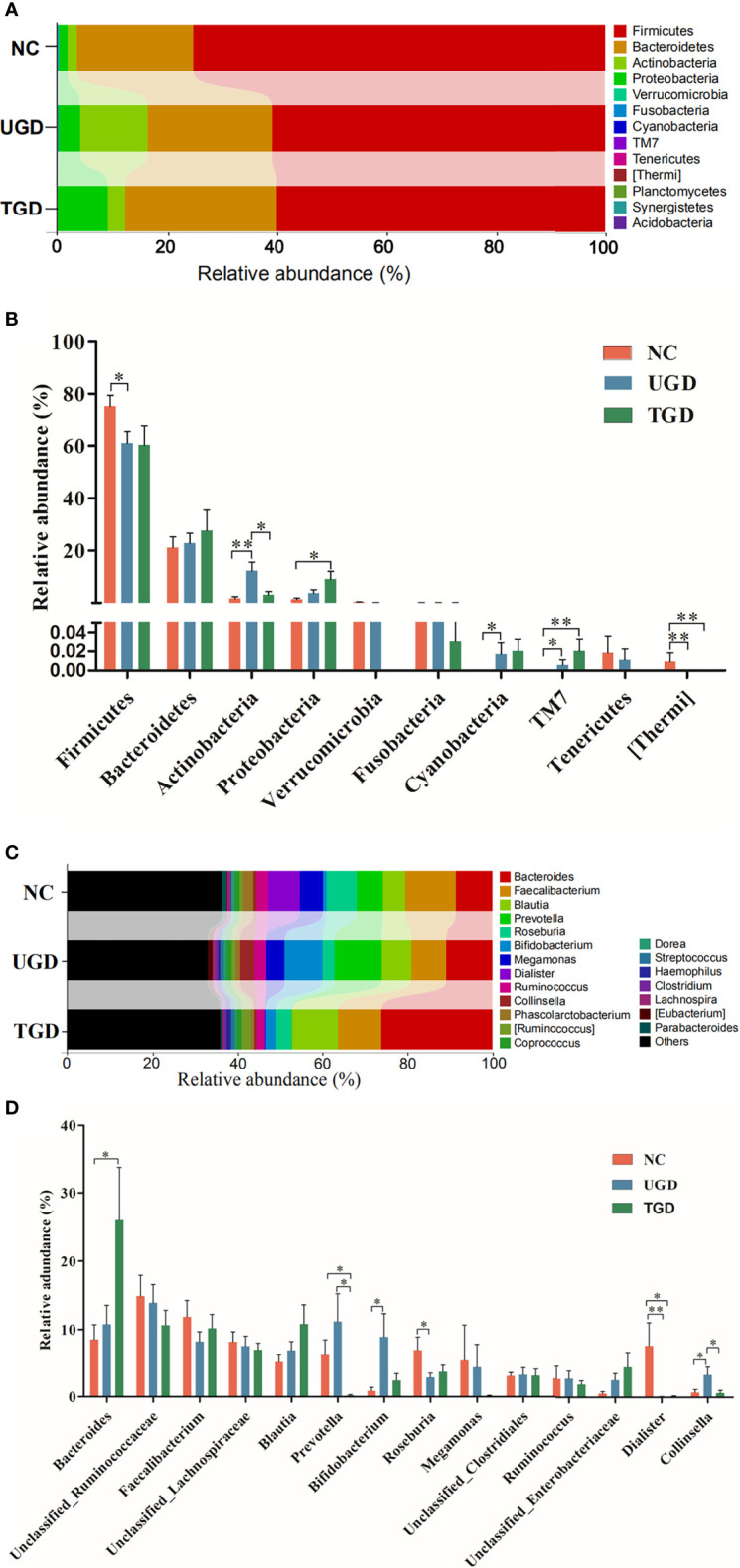
**(A)** Bar plot of the bacterial community abundance at the phylum level. **(B)** Significance of the top 10 bacterial phyla according to community abundance. **(C)** Bar plot of the bacterial community abundance at the t level. **(D)** Significance of the abundance of the top 14 bacterial communities. pairwise comparison among the three groups: *p ≤ 0.017, **p ≤ 0.001.

A total of 10 bacterial phyla were detected in the stool samples of the three groups: Firmicutes (64.7%), Bacteroidetes (23.5%), Actinobacteria (7.0%), Proteobacteria (4.5%), Verrucomicrobia (`0.1%), Fusobacteria (0.1%), Cyanobacteria (<0.05%), TM7 (<0.05%), Tenericutes (<0.05%), and [Thermi] (<0.05%). Among them, Firmicutes, Bacteroidetes, Actinobacteria, and Proteobacteria were the dominant phyla, accounting for more than 99% of the total bacteria (**Figure 5A**). Metastats analysis was used to test the differences in the sequence abundance of each taxon at the phylum level between different groups, and we found that the relative abundances of Firmicutes, Actinobacteria, Cyanobacteria, TM7, and [Thermi] between the NC group and the UGD group were different. Relative to the NC group, Actinobacteria, Cyanobacteria, and TM7 were significantly increased in the UGD group, while Firmicutes and [Thermi] were decreased (P<0.017). Relative to the NC group, Proteobacteria and TM7 were significantly increased in the TGD group, while [Thermi] was decreased (P<0.05), and the relative abundance of Actinobacteria in the UGD group was higher than that in the TGD group (P<0.017) (**Figure 5B**). Through further classification and identification at the genus level, we found that in the UGD group, the Collinsella abundance was significantly higher than those in the NC group and TGD group, the Bifidobacterium abundance was significantly higher than that in the NC group, and the Dialister and Roseburia abundances were significantly lower than those in the NC group (P<0.017). In addition, the Prevotella abundance in the TGD group was lower than those in the NC group and UGD group (P<0.017) (**Figures 5C, D**).

**Figure 6:**
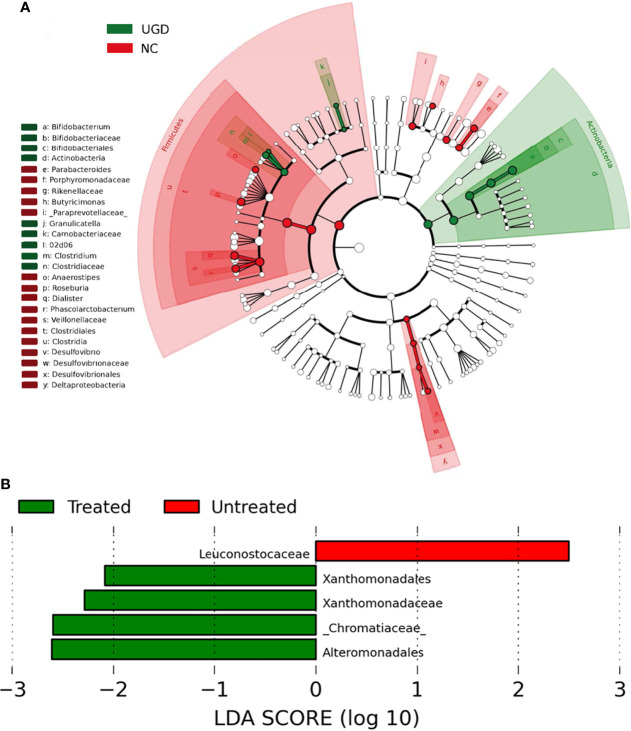
**(A)** Phylogenetic profiles of the specific bacterial taxa and predominant bacteria associated with Graves’ disease determined using the LEfSe method. **(B)** The greatest differences at the order and family levels between the UGD and TGD groups. Linear discriminant analysis, LDA score (log10), LDA score>2, *P* < 0.05.

LEfSe analysis is a recently developed method for analyzing the effect size based on linear discriminant analysis (LDA), the essence of which is to combine LDA with the nonparametric Kruskal–Wallis and Wilcoxon rank sum tests to filter key members of communities. Phylogenetic profiles of the specific bacterial taxa and predominant bacteria associated with GD at the order level between the UGD and TGD groups according to the LEfSe method showed that, relative to the NC group, Bifidobacteriaceae, Clostridiaceae, and Carnobacteriaceae showed the predominant abundance distribution in the UGD group, but Porphyromona-daceae, Rikenellaceae, Paraprevotellaceae, Veillonellaceae, and Desulfovibrionaceae showed the predominant abundance distribution in the NC group. At the genus level, Bifidobacterium, 02d06, Clostridium, and Granulicatella showed the dominant abundance distribution in the UGD group relative to the NC group, while the abundances of Parabacteroides, Butyricimonas, Anaerostipes, Roseburia, Dialister, Phoscolarctobacterium, and Desulfovibrio were significantly lower than those in the NC group (**Figure 6A**). At the order and family levels, Xanthomonadates, Xanthomonadaceae, Chromatiaceae, and Alteromonadales were enriched in the TGD group, while Leuconostocaceae was mainly enriched in the UGD group (LDA value>2, P<0.017) (**Figure 6B**).

Using Mothur software and the Metastats statistical algorithm, we performed pairwise comparisons of the differences in the sequence abundance of each taxon at the phylum and genus levels among samples. For each Metastats pairwise comparison of test samples, the corresponding P and Q values were obtained. The P value should be lower than 0.05, and the closer the P value was to zero, the stronger the power of the statistical test and the greater the statistically significant difference between samples. Since the P value will cause errors due to multiple comparison tests, the false discovery rate (FDR) should be used to correct it. The corrected P value is referred to as the Q value. The closer the Q value is to zero, the lower the probability of false positives, the greater the significance of the statistical test, and the larger the real significant difference between samples. Comparison between the NC group and the UGD group revealed that there were statistically significant differences in the abundances of 5 phyla and 21 genera (P<0.017), 4 phyla and 25 genera between the NC and the TGD group (P<0.017), and 2 phyla and 27 genera between the UGD group and the TGD group (P<0.017). The specific species abundance, variance, standard error, P value and Q value data are shown in **Tables 3**–**5**.

**Table 3:** Statistical table for the comparison of the Metastats test results among the 3 groups.

Sample	Phylum	Genus
NC-UGD	5	21
NC-TGD	4	25
UGD-TGD	2	27

The first column in the table shows the pairwise comparison groups. “Phylum” and “Genus” correspond to the number of taxa with significant differences at the phylum and genus levels in each comparison.

**Table 4:** Statistical table for the comparison of the Metastats test results among the 3 groups at the phylum level.

Phylum	UGD group			NC group						*P value*	*Q value*
	Mean	Variance	Stderr	Mean	Variance			Stderr			
[Thermi]	0	0	0	0.000056	0			0.000056		0.000061	0.001211
Actinobacteria	0.122924	0.02045	0.033706	0.017524	0.000554			0.007094		0.002997	0.029911
TM7	0.000104	0	0.000038	0.000011	0			0.000007		0.010989	0.073117
Firmicutes	0.608763	0.035459	0.044384	0.752196	0.019877			0.042509		0.043956	0.19941
Cyanobacteria	0.000232	0	0.00011	0.000028	0			0.000022		0.04995	0.19941
**Phylum**	**TGD group**			**NC group**						***P* value**	***Q* value**
	**Mean**	**Variance**	**Stderr**	**Mean**		**Variance**	**Stderr**				
[Thermi]	0	0	0	0.000056		0	0.000056			0.002123	0.020715
TM7	0.000228	0	0.000101	0.000011		0	0.000007			0.005994	0.029244
Proteobacteria	0.090661	0.009257	0.030426	0.015352		0.000187	0.004125			0.011988	0.038992
Planctomycetes	0.00003	0	0.000019	0		0	0			0.02498	0.060936
**Genus**	**UGD group**			**TGD group**						***P* value**	***Q* value**
	**Mean**	**Variance**	**Stderr**	**Mean**		**Variance**			**Stderr**		
*Actinobacteria*	0.122924	0.02045	0.033706	0.031761		0.001632			0.012774	0.015984	0.213971
*Planctomycetes*	0.000003	0	0.000003	0.00003		0			0.000019	0.024856	0.213971

Table 5Statistical table for the comparison of the Metastats test results among the 3 groups at the genus level.

**Table:** 

Genus	UGD group			NC group			*P* value	*Q* value
	Mean	Variance	Stderr	Mean	Variance	Stderr		
*Thermus*	0	0	0	0.000088	0	0.000088	0.000052	0.008961
*Slackia*	0	0	0	0.000052	0	0.000052	0.000998	0.05775
[*Prevotella*]	0	0	0	0.012881	0.001688	0.012386	0.000999	0.05775
*Pediococcus*	0.000065	0	0.00006	0	0	0	0.001743	0.075566
N09	0.000061	0	0.000036	0	0	0	0.002982	0.086625
*Dialister*	0.001242	0.000008	0.000672	0.111191	0.023758	0.046474	0.002997	0.086625
*Oxalobacter*	0.000005	0	0.000005	0.000068	0	0.000059	0.005383	0.133352
02d06	0.000211	0	0.000098	0.000014	0	0.000014	0.006993	0.13852
*Mycoplana*	0	0	0	0.000044	0	0.000044	0.007189	0.13852
*Enterobacter*	0.00005	0	0.000036	0	0	0	0.009297	0.161237
*Anaerostipes*	0.000507	0	0.000126	0.005909	0.000086	0.002796	0.010989	0.173249
*Bulleidia*	0.000037	0	0.00002	0	0	0	0.029365	0.363363
*Weissella*	0.000035	0	0.000027	0	0	0	0.029365	0.363363
*Bifidobacterium*	0.138593	0.04719	0.051202	0.013941	0.000876	0.008924	0.02997	0.363363
*Gemella*	0.000045	0	0.000029	0	0	0	0.031429	0.363363
*Collinsella*	0.048576	0.005073	0.016788	0.009258	0.000274	0.00499	0.036963	0.384998
*Butyricimonas*	0.000074	0	0.000069	0.001439	0.000004	0.000629	0.038961	0.384998
*Roseburia*	0.040829	0.001794	0.009983	0.106727	0.007904	0.026806	0.03996	0.384998
*Corynebacterium*	0	0	0	0.000121	0	0.000085	0.044955	0.387748
*Odoribacter*	0.000064	0	0.00003	0.001149	0.000003	0.000559	0.044955	0.387748
*Granulicatella*	0.000192	0	0.000116	0	0	0	0.046953	0.387748

**Table:** 

Genus	TGD group			NC group			*P* value	*Q* value
	Mean	Variance	Stderr	Mean	Variance	Stderr		
*Burkholderia*	0.000367	0	0.000151	0	0	0	0.000999	0.028661
*Campylobacter*	0.000125	0	0.000125	0	0	0	0.000999	0.028661
*Lactobacillus*	0.019577	0.003797	0.019485	0	0	0	0.000999	0.028661
*Sphingobium*	0.000112	0	0.000087	0	0	0	0.000999	0.028661
*Microbacterium*	0.000086	0	0.000042	0	0	0	0.001654	0.032755
*Thermus*	0	0	0	0.000088	0	0.000088	0.001982	0.032755
*Granulicatella*	0.00011	0	0.00005	0	0	0	0.001998	0.032755
*Bulleidia*	0.000078	0	0.000051	0	0	0	0.00337	0.048348
Lactococcus	0.000075	0	0.000042	0	0	0	0.006866	0.087553
Erythrobacter	0.000104	0	0.000053	0	0	0	0.007992	0.091714
Veillonella	0.012495	0.000587	0.00766	0.000342	0	0.000167	0.011988	0.125064
*Dialister*	0.002214	0.000021	0.001453	0.111191	0.023758	0.046474	0.014985	0.128963
*Prevotella*	0.002641	0.000034	0.001839	0.089928	0.011385	0.032171	0.014985	0.128963
Slackia	0	0	0	0.000052	0	0.000052	0.015733	0.128963
Aminobacter	0.000132	0	0.000054	0.000009	0	0.000009	0.020979	0.160499
Ochrobactrum	0.007374	0.000093	0.003044	0.001131	0.000004	0.000601	0.027972	0.184701
Limnohabitans	0.000054	0	0.000038	0	0	0	0.028498	0.184701
Methylobacterium	0.000143	0	0.000067	0.000008	0	0.000008	0.028971	0.184701
Sphingomonas	0.000363	0	0.000181	0.000036	0	0.000022	0.035964	0.217217
Ralstonia	0.000237	0	0.000122	0.000022	0	0.000015	0.03996	0.218366
*Sediminibacterium*	0.004056	0.000032	0.001776	0.000619	0.000001	0.000338	0.03996	0.218366
*Brevundimonas*	0.000413	0	0.000164	0.000063	0	0.000043	0.043956	0.229285
*Agrobacterium*	0.003971	0.000026	0.001604	0.000702	0.000001	0.000353	0.047952	0.229285
*Amycolatopsis*	0.000154	0	0.000074	0.000023	0	0.000016	0.04995	0.229285
*Bacteroides*	0.326728	0.069912	0.083614	0.130101	0.009706	0.029705	0.04995	0.229285

**Table:** 

Genus	UGD group			TGD group			*P* value	*Q* value
	Mean	Variance	Stderr	Mean	Variance	Stderr		
*Campylobacter*	0	0	0	0.000125	0	0.000125	0.000999	0.058444
*Mycoplana*	0	0	0	0.000144	0	0.00008	0.000999	0.058444
*Acinetobacter*	0.000229	0	0.000128	0.003325	0.000016	0.001279	0.001998	0.058444
*Burkholderia*	0.00001	0	0.00001	0.000367	0	0.000151	0.001998	0.058444
N09	0.000061	0	0.000036	0	0	0	0.003293	0.065931
*Pediococcus*	0.000065	0	0.00006	0	0	0	0.003381	0.065931
*Oscillospira*	0.000883	0.000001	0.000285	0.005351	0.000025	0.001569	0.004995	0.074383
*Anaerotruncus*	0	0	0	0.000049	0	0.000026	0.006406	0.074383
*Devosia*	0	0	0	0.000046	0	0.000027	0.006406	0.074383
*Limnohabitans*	0	0	0	0.000054	0	0.000038	0.006406	0.074383
*Sediminibacterium*	0.000319	0.000001	0.000199	0.004056	0.000032	0.001776	0.006993	0.074383
*Ochrobactrum*	0.000663	0.000002	0.000353	0.007374	0.000093	0.003044	0.007992	0.077925
*Enterobacter*	0.00005	0	0.000036	0	0	0	0.009672	0.083491
*Brevundimonas*	0.000031	0	0.000015	0.000413	0	0.000164	0.00999	0.083491
*Novosphingobium*	0.000005	0	0.000005	0.000061	0	0.000039	0.011232	0.087615
*Lactococcus*	0.000009	0	0.000009	0.000075	0	0.000042	0.014247	0.104186
*Agrobacterium*	0.000385	0.000001	0.000239	0.003971	0.000026	0.001604	0.016983	0.108319
*Acidovorax*	0	0	0	0.000045	0	0.000038	0.01759	0.108319
*Delftia*	0	0	0	0.000042	0	0.000029	0.01759	0.108319
*Prevotella*	0.146658	0.047289	0.051256	0.002641	0.000034	0.001839	0.020979	0.122732
*Phyllobacterium*	0.00001	0	0.000007	0.000186	0	0.000072	0.022977	0.12802
*Oribacterium*	0.000004	0	0.000004	0.000049	0	0.000033	0.02677	0.142298
*Sphingomonas*	0.00003	0	0.000016	0.000363	0	0.000181	0.027972	0.142298
*Collinsella*	0.048576	0.005073	0.016788	0.009412	0.000377	0.006136	0.030969	0.149616
*Ralstonia*	0.000018	0	0.00001	0.000237	0	0.000122	0.031968	0.149616
*Eggerthella*	0.000153	0	0.000067	0.001267	0.000003	0.000575	0.034965	0.157349
*Herbaspirillum*	0	0	0	0.000033	0	0.000026	0.0483	0.209308

### 3.7 Correlation Analysis Between Bifidobacterium and Serum Thyroid Indicators

**Figure 7:**
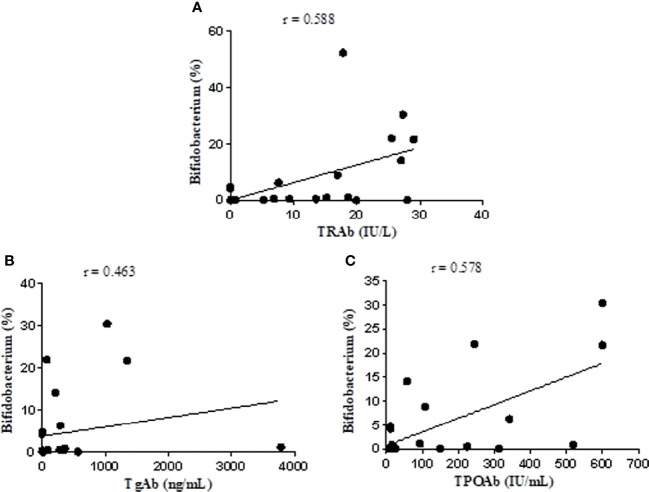
Correlation analysis of *Bifidobacterium* with TRAb, TgAb, and TPOAb based on the data from the NC and UGD groups. **(A)** Bifidobacterium and TRAb. **(B)** Bifidobacterium and TgAb. **(C)** Bifidobacterium and TPOAb.

The Spearman rank test was used to analyze the correlation of the relative abundance of Bifidobacterium with FT3, FT4, TSH, TRAb, TgAb, and TPOAb levels based on data from the NC and UGD groups. The results showed that Bifidobacterium was positively correlated with TRAb (r=0.588, P=0.002), TgAb (r=0.463, P=0.023), and TPOAb (r=0.578, P=0.002) (**Figure 7**) but not with FT3, FT4 or TSH (r=0.028, 0.023 and 0.105, respectively; P>0.05).

## 4 Discussion

The relationship between the intestinal flora and autoimmune-related diseases has become a research focus in recent years. The pathogenesis of GD has not yet been fully clarified, the recurrence rate of the disease is high, and the outcome after treatment varies greatly among individuals. However, it has been proven that based on genetic predisposition, immune dysfunction caused by environmental factors is an important component of its pathogenesis, and environmental risk factors are closely related to the composition and structure of the intestinal flora. In the past, the understanding of the intestinal flora mainly relied on traditional culture methods, microscopy, and the biochemical characteristics of the bacteria. However, with the development of 16S rRNA gene high-throughput sequencing technology in recent years, this approach has been widely used in the study of intestinal flora due to its advantages, such as deep sequencing and high accuracy (Goldfeder et al., 2017). The role of intestinal microbiota in the pathogenesis of GD has only been discovered in recent years based on mouse models and clinical studies. GD therapeutic drugs (such as antithyroid drugs and glucocorticoids) can also change the composition of the microbiota (Zhao et al., 2020; Chen et al., 2021). In this study, the composition and structure of the intestinal flora of GD patients were analyzed and compared with those of a healthy population, and the results showed decreased diversity of the intestinal flora in GD patients. This finding was consistent with the findings of Ishaq HM et al., indicating that the biological diversity of the intestinal flora of GD patients is reduced (Ishaq et al., 2018).

Metastats analysis showed that at the phylum level, Cyanobacteria, TM7, and Actinobacteria abundances were significantly higher in newly diagnosed GD patients than in healthy people and that the Actinobacteria abundance decreased after treatment with methimazole. Further classification and identification at the genus level revealed that the species within Actinobacteria that caused significant differences at the genus level in newly diagnosed GD patients were Bifidobacterium and Collinsella. However, the Firmicutes phylum and the [Thermi] phylum of newly diagnosed GD patients showed lower abundances than were observed in healthy people. The Proteobacteria phylum and the TM7 phylum showed a gradual increasing trend in healthy people, newly diagnosed GD patients, and GD patients after methimazole treatment, and their abundances were significantly higher in GD patients after methimazole treatment than in healthy people. Among these groups, the classification and identification of Bacteroidetes at the taxonomic level of genera showed that the Prevotella abundance was significantly higher in newly diagnosed GD patients than in healthy people. Roseburia showed significantly lower abundance in newly diagnosed GD patients than in healthy people, and its abundance increased after treatment with methimazole tablets. The Dialister abundance was significantly higher in healthy people than in GD patients both before and after treatment with methimazole. This finding is consistent with the findings of Ishaq HM et al., who found that compared with healthy people, GD patients showed a significant decrease in Dialister and a significant increase in Prevotella (Ishaq et al., 2018). According to a recently published review, several taxa were reported to be differentially represented in GD patients, including Prevotellaceae and Veillonellaceae at the family level and Bacteroides, Lactobacillus, Prevotella, and Veillonella at the genus level (Hou et al., 2021).

Relevant studies have shown that Th17 and Treg cells (Th17/Treg axis) may be involved in the pathogenesis of GD (Klatka et al., 2014; Qin et al., 2017). Th17 cells account for approximately 1% of CD4+ T cells and exert a strong specific proinflammatory effect on autoantigens, which may cause severe autoimmune reactions (Hundorfean et al., 2012). Thyroid tissue is the main target organ of the inflammatory process of autoimmune thyroid disease (AITD). Recently, it has been found that as the disease progresses, Th17 cells migrate to the inflammation site and induce the production of proinflammatory cytokines (IL-6, IL-1, and TNFα) and chemokines to induce Th1 cells to aggravate the inflammatory response in thyroid tissue (Dardalhon et al., 2008). Relative to healthy people, one study showed that the levels of Treg cells in the thyroid tissue and peripheral blood of AITD patients were reduced and that the level of CD4+CD25+ Treg cells in untreated patients was negatively correlated with the concentration of TPOAb (Bossowski et al., 2012). Treg cells can inhibit autoreactive T cells and B cells and maintain autoimmune tolerance through the secretion of IL-10, TGF-β and other regulatory factors (Pyzik et al., 2015; Kristensen, 2016). Our previous study also found that IL-17 in GD patients was significantly higher than that in the control group (Yang et al., 2021).

Studies have shown that Roseburia is associated with intestinal health, inflammatory bowel disease, irritable bowel syndrome, colon cancer, and coronary atherosclerosis. In patients with ulcerative colitis, the abundance of butyrate-producing Roseburia bacteria is decreased, which is associated with decreasing concentrations of short-chain fatty acids (SCFAs) (Karlsson et al., 2012; Kumari et al., 2013; Machiels et al., 2015). Excitingly, we also found in this study that the abundance of the beneficial intestinal bacterium Roseburia was significantly reduced in untreated GD patients and was slightly increased after treatment with methimazole tablets. Roseburia can produce acetic acid, butyric acid, and other SCFAs by breaking down indigestible carbohydrates (Louis and Flint, 2009), and it can induce the production of Treg cells and promote their function, probably by releasing SCFAs in the intestine (Arpaia et al., 2013). Butyrate can increase the production of Treg cells and IL-10 by activating the cell receptor HCA2. Propionate and acetate can promote the production of peripheral Tregs. In particular, butyrate can enhance the acetylation of histone H3 in the conserved noncoding sequence of the FOX-P3 gene intron, promoting the generation of Treg cells outside the thymus (Smith et al., 2013). SCFAs can also promote the synthesis of intestinal mucosins and the secretion of intestinal epithelial cell mucus, thereby repairing and improving the function of the intestinal mucosal barrier, inhibiting the secretion of proinflammatory factors, increasing the production of the anti-inflammatory factor IL-10, and activating Treg cell activity (Miao et al., 2016; Shen et al., 2019). ATD treatment reduced short-chain fatty acid (SCFA)-producing bacteria, including Faecalibacterium, Ruminococcaceae, Lactobacillus, and Blautia (Su et al., 2020). In this study, we found that the Roseburia abundance was lower in GD patients both before and after methimazole tablet treatment than in healthy people. Therefore, we speculated that, first, the amount of SCFAs produced by Roseburia was significantly reduced and that the number of induced differentiated Treg cells and, hence, the release of anti-inflammatory cytokines were reduced accordingly. Second, we hypothesized that the reduction in the synthesis of intestinal mucosin and in the secretion of intestinal epithelial cell mucus leads to a decrease in the defense function of intestinal epithelial cells and that the increase in intestinal mucosal permeability contributes to the translocation of toxins and antigens produced by bacteria and even the bacteria themselves, thereby causing or aggravating GD.

Under normal conditions, the intestinal mucosal barrier can prevent the translocation of microorganisms and their harmful metabolites from the intestinal lumen to the blood, but when intestinal homeostasis is imbalanced or damaged, it may lead to “leaky intestinal syndrome”, including an increase in permeability, the activation of the nonspecific immune system and the promotion of low-grade inflammation. Therefore, it is essential to maintain intestinal homeostasis (König et al., 2016). This study showed that the Collinsella abundance increased significantly in untreated GD patients and decreased significantly after treatment with methimazole tablets. The pathogenic bacteria of this genus may induce and aggravate autoimmune diseases. Their abundance in the intestinal flora of patients with spondylitis is significantly increased and is related to the occurrence of the disease (Mu et al., 2017; Chen et al., 2019). Studies have shown that the abundance of Collinsella is related to the production of the proinflammatory factor IL-17 (Chen et al., 2016). Reducing the expression of the tight junction protein ZO-1 in epithelial cells induces increased expression of IL-17A and RORα and the chemokines CXCL1 and CXCL5; moreover, RORα, CXCL1, and CXCL5 can regulate the production of IL-17 (Yang et al., 2008; Nouailles et al., 2014) and increase the permeability of the intestinal mucosal barrier. Therefore, we speculated that Collinsella may participate in the pathogenesis of GD by changing the permeability of the intestinal mucosa and inducing the expression of IL-17 and other cytokines.

In this study, another significantly increased bacterial genus identified in untreated GD patients was Bifidobacterium, which was in line with the results reported in the literature (Song et al., 2019). After treatment with methimazole tablets, however, its abundance decreased. The relative abundance of this bacterium was positively correlated with the levels of TRAb, TgAb, and TPOAb (r=0.588, 0.463, and 0.578, respectively; P values were all < 0.05). These findings have not been reported by other scholars. Some people believe that certain strains of Bifidobacterium act as probiotics that can have a beneficial effect on the body, and these bacteria have been used to treat acute infectious diarrhea, antibiotic-related diarrhea, ulcerative colitis, irritable bowel syndrome, and other diseases (Tamaki et al., 2016; Sharif et al., 2017; Xu et al., 2017). Microbes are not absolutely “good” or “bad”. Bifidobacterium and Lactobacillus are considered probiotics in most cases, and their numbers are often reduced in disease states, but there may be exceptions in individual diseases. It has been reported that taking probiotics can cause inflammation through the production of Th1 cells, leading to a negative impact on autoimmune diseases mediated by Th1 cells (Delcenserie et al., 2008). Th1 cell activation can directly damage thyroid follicular cells. In a rat model study, the mechanism of the Th1 cell-mediated destruction of thyroid cells was shown to involve IL-12, IFN-γ and TNF-α (Luty et al., 2019). Studies have shown that certain strains of Bifidobacterium and Lactobacillus may be involved in triggering AITD through molecular simulation mechanisms. Some specific antibodies against Bifidobacterium antigens have been detected in the serum of AITD patients. Certain strains of Bifidobacterium and Lactobacillus share the same amino acid sequences of thyroid peroxidase (TPO) and Tg and can selectively bind to autoantibodies, which may participate in triggering AITD through a molecular simulation mechanism (Kiseleva et al., 2011). The specific mechanism of how methimazole treatment affects intestinal flora is unclear, and in-depth research remains to be conducted to reveal this process. At present, we speculate that how methimazole affects the parameters related to the intestinal flora and thyroid function and the relationship between them may be related to the following factors (1): intestinal flora (such as Bifidobacterium, Lactobacillus, and Prevotella) can simulate thyroid peroxidase (TPO), which is an important enzyme for thyroid hormone synthesis (2). Methimazole can inhibit TPO, inhibit the synthesis of T3 and T4, reduce the release of thyroid hormone into the blood, and reduce the level of thyroid stimulating antibody in the blood. After treatment with methimazole, Bifidobacterium decreased significantly. GD is an AITD (3). Methimazole treatment reduced short-chain fatty acid (SCFA)-producing bacteria, such as Roseburia, which can induce the production of Treg cells in the intestine and promote their function (4). Some intestinal flora can also participate in the metabolism of methimazole and can change the activity or toxicity of drugs.

According to this study, patients with newly diagnosed GD mainly showed an increase in some pathogenic bacteria, such as Collinsella and Bifidobacterium, while beneficial bacteria producing short-chain fatty acids, such as Roseburia, decreased significantly after treatment; the change in the Bifidobacterium abundance in patients with newly diagnosed GD was positively correlated with TRAb, TGAb and TPOAb. These changes are suggested to be related to the immune mechanism of GD. Moreover, the data for several batches of specimens we collected were highly reproducible, and the result is that Bifidobacterium increases in the feces of GD patients. Therefore, it is reasonable to believe that the flora may affect the progression of GD, especially Bifidobacterium, but what kind of subgroup or structure of Bifidobacterium affects the progress of GD may require further screening, identification and extraction of specific special flora for repeated verification by gavage or intestinal flora transplantation in sterile mice.

There are some limitations of this study. For example, the number of GD samples examined was relatively small, and the fecal flora cannot fully reflect the intestinal flora colonizing the intestinal mucosa. These limitations further require the expansion of the sample size and the examination of the role of the intestinal flora in the pathogenesis of GD at the mechanistic level.

The biological diversity of the intestinal flora is reduced in GD patients. There are differences in the intestinal flora between normal people and GD patients and between GD patients before and after methimazole treatment, mainly manifesting as changes in the abundance of pathogenic bacteria, such as Collinsella members, and the abundance of short-chain fatty acid-producing beneficial bacteria, such as Roseburia. The change in Bifidobacterium abundance is positively correlated with the levels of TRAb, TgAb, and TPOAb, suggesting that this may be involved in the immune mechanism of GD. Therefore, we speculate that an intestinal flora disorder might be involved in the pathogenesis of GD.

## Data Availability Statement

The original contributions presented in the study are included in the article/supplementary material. Further inquiries can be directed to the corresponding author.

## Ethics Statement

The studies involving human participants were reviewed and approved by the Ethics Committee of the Affiliated Hospital of Zunyi Medical University (2018) 1-145. The patients/participants provided their written informed consent to participate in this study.

## Author Contributions

MY designed the research. XZ, YW, RZ, QG, BY, BS, and QY performed the research. ZY, JL, BZ, and MZ analyzed the data. MY wrote the original draft and made the figures. All authors contributed to the drafting or editing of parts of the manuscript and approved the final version of the manuscript.

## Funding

The study was supported by the Shanghai Municipal Health Commission (No. 202140345) and the Medical Key Faculty Foundation of Shanghai (grant no., ZK2019B15).

## Conflict of Interest

The authors declare that the research was conducted in the absence of any commercial or financial relationships that could be construed as a potential conflict of interest.

## Publisher’s Note

All claims expressed in this article are solely those of the authors and do not necessarily represent those of their affiliated organizations, or those of the publisher, the editors and the reviewers. Any product that may be evaluated in this article, or claim that may be made by its manufacturer, is not guaranteed or endorsed by the publisher.
